# Clinical and ultrasound-based optimization of post-FNA management decisions in Bethesda III/IV thyroid nodules: a retrospective study

**DOI:** 10.3389/fendo.2026.1807083

**Published:** 2026-05-21

**Authors:** Junmei Wang, Songfeng Jia, Jianling Li, Qiru Zhang, Hua Liu, Mengyang Zhao

**Affiliations:** 1 Department of Ultrasound II, Xinxiang Central Hospital, Xinxiang, Henan, China; 2 Department of Magnetic Resonance Imaging, Xinxiang Second People’s Hospital, Xinxiang, Henan, China

**Keywords:** Bethesda classification, decision curve analysis, fine-needle aspiration, multivariable prediction model, thyroid nodule

## Abstract

**Background:**

Indeterminate thyroid cytology classified as Bethesda III or IV represents a persistent clinical dilemma, with substantial uncertainty regarding malignancy risk and frequent reliance on diagnostic surgery. Optimizing post–fine-needle aspiration (FNA) management using readily available clinical and ultrasonographic information may reduce unnecessary surgical intervention while preserving oncologic safety.

**Methods:**

We conducted a single-center retrospective cohort study including 206 adult patients with Bethesda III or IV thyroid nodules evaluated. Clinical characteristics, biochemical parameters, and standardized ultrasound features were extracted. Malignancy was defined by surgical histopathology or a prespecified composite reference standard. Univariable and multivariable logistic regression analyses were performed to identify independent predictors of malignancy. Model discrimination and calibration were assessed using the area under the receiver operating characteristic curve (AUC), Brier score, and calibration slope. The diagnostic yield of repeat FNA was evaluated, and an optimized post-FNA management pathway was developed and compared with observed clinical practice using paired analyses and decision curve analysis.

**Results:**

Malignancy was confirmed in 87 of 206 nodules (42.2%). In multivariable analysis, lower free thyroxine levels (adjusted odds ratio [OR] 0.63, 95% confidence interval [CI] 0.48–0.84), taller-than-wide shape (OR 3.69, 95% CI 1.21–11.27), marked hypoechogenicity (OR 3.12, 95% CI 1.01–9.64), punctate echogenic foci (OR 2.01, 95% CI 1.01–4.89), and suspicious cervical lymph nodes (OR 4.31, 95% CI 1.10–16.90) were independently associated with malignancy. The multivariable model demonstrated good discrimination, with an apparent AUC of 0.875 and an optimism-corrected AUC of 0.798, along with acceptable calibration (Brier score 0.183). Repeat FNA was performed in 73 patients (35.4%), yielding actionable cytologic reclassification in 53.4%, including upgrading to Bethesda V/VI in 9.6%. Compared with observed practice, the optimized pathway reduced the proportion of nodules assigned to surgery (70.9% *vs* 57.8%) and unnecessary surgery among all nodules (38.3% *vs* 29.1%), while maintaining similar use of repeat FNA. When restricted to operated nodules, the corresponding rate of unnecessary surgery was 54.1%. Decision curve analysis showed superior net clinical benefit of the optimized pathway across clinically relevant risk thresholds.

**Conclusions:**

Integrating conventional clinical and ultrasound features provides an effective framework for post-FNA risk stratification in Bethesda III and IV thyroid nodules. An optimized management pathway based on this approach may reduce unnecessary surgery while maintaining appropriate malignancy detection, supporting more individualized and proportionate clinical decision-making.

## Introduction

Thyroid nodules are highly prevalent in clinical practice, and the widespread use of high-resolution ultrasonography has further increased incidental detection in both symptomatic and asymptomatic populations (1). Fine-needle aspiration (FNA) remains the cornerstone for preoperative risk stratification, yet a substantial proportion of aspirates fall into indeterminate cytology categories in which benignity or malignancy cannot be confidently established (2). In contemporary practice, indeterminate nodules collectively account for roughly one-fifth of all thyroid FNAs, with pooled estimates around 19.7 percent overall, including approximately 9.6 percent classified as Bethesda category III (atypia of undetermined significance or follicular lesion of undetermined significance) and 10.1 percent as Bethesda category IV (follicular neoplasm or suspicious for a follicular neoplasm) (2–4). These categories represent a diagnostic gray zone rather than a definitive cancer diagnosis, and their implied risk of malignancy varies widely by institution, case mix, and evolving histopathologic definitions (5). The 2017 revision of The Bethesda System updated expected malignancy risks and emphasized the impact of entities such as noninvasive follicular thyroid neoplasm with papillary-like nuclear features, with commonly cited malignancy risk ranges of approximately 10 to 30 percent for Bethesda III and 25 to 40 percent for Bethesda IV when noninvasive follicular thyroid neoplasm with papillary-like nuclear features is included among malignant outcomes (6). Despite standardized reporting, real-world malignancy rates in resected Bethesda III and IV nodules can exceed the implied Bethesda estimates in large datasets, underscoring persistent uncertainty and selection effects in surgical cohorts (7). This variability is further influenced by evolving histopathologic definitions, particularly the classification of noninvasive follicular thyroid neoplasm with papillary-like nuclear features. For clinicians, this uncertainty is not merely academic. Indeterminate cytology frequently triggers downstream diagnostic procedures and operations, exposing many patients with ultimately benign disease to avoidable costs, surgical risks, and long-term consequences such as lifelong thyroid hormone replacement and potential voice or calcium-related complications (8).

The critical clinical challenge, therefore, is not whether to perform an initial FNA, but how to manage nodules after an indeterminate result. Current guidelines support individualized post-FNA decision-making that integrates clinical risk factors, sonographic risk pattern, and patient preferences, while acknowledging low-quality evidence and substantial practice variation (9). For Bethesda III nodules, repeat FNA, molecular testing, active surveillance, or diagnostic surgical excision are each considered reasonable depending on the overall risk profile and the degree of concern raised by ultrasonographic features (10). For Bethesda IV nodules, diagnostic lobectomy has traditionally been favored because cytology cannot distinguish follicular adenoma from follicular carcinoma without capsular or vascular invasion assessment, yet even within Bethesda IV the malignancy risk remains heterogeneous and increasingly influenced by imaging pattern and clinical context (6, 9). In parallel, molecular classifiers and mutation panels have been developed to refine risk stratification and reduce unnecessary diagnostic surgery, but their performance, cost-effectiveness, and generalizability vary across settings, and access remains uneven across healthcare systems (11). Moreover, even where molecular testing is available, clinicians still must decide which patients can be safely observed, which require prompt rebiopsy, and which benefit from surgery despite nondiagnostic or equivocal ancillary testing. The practical reality is that many institutions rely primarily on clinical and ultrasound features to guide post-FNA management, especially when molecular testing is unavailable, unaffordable, or not routinely reimbursed (9, 12). Yet, the evidence base for how best to combine these readily available factors into a coherent, reproducible post-FNA pathway for Bethesda III and IV nodules remains incomplete.

Several gaps limit current decision-making. First, most studies of indeterminate nodules have emphasized prediction of malignancy among surgically treated patients, which can inflate malignancy estimates and does not directly address whether a given management choice was necessary or avoidable (13). Second, although sonographic risk stratification systems are widely used before biopsy, their role after indeterminate cytology is less clearly operationalized, and many real-world pathways do not explicitly define when repeat FNA is expected to provide meaningful diagnostic gain (14). Third, the diagnostic yield of repeat FNA in Bethesda III and IV nodules is variable, and the incremental value of rebiopsy likely depends on ultrasound pattern, nodule size, patient age, symptom burden, and comorbidities (15). Finally, the clinical outcome that matters most at the point of decision is not only malignancy detection, but also the avoidance of unnecessary surgery and the preservation of safe cancer detection. In this context, a pragmatic framework is needed that quantifies tradeoffs across management options, clarifies how repeat FNA contributes to risk resolution, and identifies patient and ultrasound profiles in which surgery is more likely to be diagnostic rather than therapeutic. Importantly, the increasing recognition of indolent or low-risk thyroid neoplasia further elevates the value of management strategies that minimize overtreatment without compromising oncologic safety (12, 15, 16).

To address these needs, we performed a retrospective study to optimize post-FNA management decisions for Bethesda III and IV thyroid nodules using clinical context and ultrasound features that are routinely available in real-world practice. We aimed to answer three clinically actionable questions. First, which clinical and ultrasonographic factors are most strongly associated with malignant outcomes in Bethesda III and IV nodules within routine care. Second, what is the diagnostic gain of repeat FNA following an initial indeterminate result, including the proportion of nodules that are reclassified into definitive benign or malignant categories after rebiopsy. Third, what proportion of surgical procedures performed after indeterminate cytology represent potentially unnecessary surgery, defined by benign final pathology, and how these events distribute across clinical and ultrasound risk strata. Building on these analyses, we sought to propose an evidence-informed, clinically implementable pathway that helps clinicians choose between repeat biopsy, surveillance, and surgery after indeterminate cytology. We hypothesized that integrating clinical variables with ultrasound risk features would provide more actionable post-FNA stratification than either domain alone, thereby improving the alignment between management intensity and true malignancy risk, increasing the efficiency of repeat FNA when it is likely to be informative, and reducing avoidable diagnostic surgery while maintaining appropriate oncologic vigilance.

## Methods

### Study design and reporting framework

This single-center retrospective cohort study evaluated and optimized management decisions after an indeterminate thyroid FNA result. The primary clinical decision point was the next step after an index Bethesda III (AUS/FLUS) or Bethesda IV (FN/SFN) cytology result, including repeat FNA, diagnostic surgery without repeat FNA, or active ultrasound surveillance. The manuscript was prepared in accordance with STROBE guidance for observational studies; when prediction modeling elements were used, TRIPOD-aligned reporting items for model development and internal validation were followed.

### Ethics

The study was conducted in accordance with the Declaration of Helsinki. Written informed consent was waived because the analysis used routinely collected clinical data and all records were de-identified prior to analysis.

### Setting, study period, and cohort assembly

We screened consecutive adult patients who underwent ultrasound-guided thyroid FNA at the study hospital between January 1, 2022 and January 31, 2024. Patients were eligible if the index cytology of the target nodule was reported as Bethesda III or Bethesda IV. The unit of analysis was the patient-level index nodule. To ensure analytic independence, only one index nodule per patient was included. If multiple nodules met eligibility criteria, the nodule with the highest ultrasound risk category was selected; if categories were identical, the largest nodule by maximal diameter was selected. The final analytic cohort comprised 206 patients with 206 index nodules. Follow-up data were censored on January 31, 2026.

### Data sources and data linkage

Cases and covariates were identified by deterministic linkage across the electronic medical record, the ultrasound reporting and image archiving system, the cytology registry, and the surgical pathology database. Linkage was performed using encounter identifiers, specimen accession numbers, examination dates, laterality, and anatomical descriptors. Records were reconciled by cross-checking lobe or isthmus location, maximal diameter, and contemporaneous procedural notes to minimize mismatches.

### Eligibility criteria

Eligibility criteria were prespecified before data extraction. Inclusion criteria were: (1) age ≥18 years; (2) a thyroid nodule evaluated by high-resolution ultrasound; (3) ultrasound-guided FNA performed during the study period with an index cytology diagnosis of Bethesda III or Bethesda IV; (4) availability of ultrasound descriptors or archived images sufficient for standardized feature abstraction; and (5) ascertainable follow-up enabling reference outcome adjudication. Exclusion criteria were: (1) prior thyroid cancer, prior thyroid surgery, or prior therapeutic neck irradiation before the index evaluation; (2) non-thyroid neck lesions miscoded as thyroid nodules; (3) purely cystic nodules without a solid component; (4) ablation or other definitive intervention performed before outcome adjudication; (5) missing cytology documentation required for Bethesda assignment; or (6) insufficient follow-up for non-operated nodules to determine the reference outcome.

### Clinical variables

Clinical variables were extracted from electronic records, including age, sex, symptom status (incidental finding vs palpable or symptomatic nodule), family history of thyroid cancer, history of therapeutic or occupational radiation exposure, autoimmune thyroiditis context, and multinodular thyroid status. Thyroid-stimulating hormone and free thyroxine were recorded if measured within 30 days before or after the index FNA; when multiple results were available, the value closest to the index FNA date was used.

### Ultrasound acquisition and feature definitions

Pre-FNA ultrasound examinations were performed on high-resolution ultrasound systems using 5–14 MHz linear-array transducers by credentialed sonographers following departmental protocols. All ultrasound examinations were performed by experienced ultrasound physicians with formal training in thyroid imaging, following standardized departmental protocols to ensure consistency of image acquisition and interpretation. Ultrasound features were abstracted from structured reports and archived images using a prespecified data dictionary. Core features included maximal diameter, lobe or isthmus location, composition (solid or almost solid, mixed cystic–solid, or spongiform), echogenicity (isoechoic or hyperechoic, hypoechoic, or markedly hypoechoic), shape on transverse view (taller-than-wide vs wider-than-tall), margin characteristics (smooth, ill-defined, lobulated or irregular, or suspected extrathyroidal extension), echogenic foci (none, macrocalcifications, rim calcifications, or punctate echogenic foci), and suspicious cervical lymph nodes.

Where sufficient information was available, nodules were also categorized using ACR TI-RADS (2017) and ATA (2015) ultrasound patterns by applying published definitions and size-based thresholds. We acknowledge that more recent updates to ATA guidance have been proposed; however, the 2015 ATA framework was applied in this study to ensure consistency with the study period and the available clinical documentation. For quality control, two trained reviewers independently abstracted ultrasound features for a random 10% subset of cases; discrepancies were resolved by consensus with a senior reviewer, and kappa statistics were calculated for key categorical features.

### Ultrasound-guided FNA procedure and cytology processing

Ultrasound-guided FNA was performed in an outpatient setting by operators experienced in thyroid interventions. After antiseptic skin preparation and sterile draping, local anesthesia with 1% lidocaine was administered subcutaneously at the operator’s discretion. Under real-time ultrasound guidance, aspiration was performed using a 23-gauge needle attached to a syringe. A transisthmic or direct approach was selected according to nodule location and surrounding vascular anatomy, with continuous visualization of the needle tip. Operators prioritized sampling solid components and avoided cystic or necrotic areas when feasible. Two to three needle passes were routinely obtained, with additional passes when adequacy was uncertain. Rapid on-site evaluation was not routinely available.

Specimens were prepared as direct smears for Papanicolaou staining (Merck KGaA, Darmstadt, Germany) and Diff-Quik staining (Siemens Healthineers, Erlangen, Germany). Residual material was processed using a liquid-based cytology system (ThinPrep, Hologic, Marlborough, MA, USA) according to routine laboratory workflow. Cytology was reported by board-certified cytopathologists using the Bethesda System for Reporting Thyroid Cytopathology. Adequacy criteria were applied according to Bethesda guidance.

### Post-FNA management pathways

Post-FNA management was defined according to the actual clinical course following the index Bethesda III or IV result. The initial post-FNA strategy was categorized as: (1) repeat FNA as the next step; (2) diagnostic surgery without repeat FNA; or (3) active surveillance with serial ultrasound. Repeat FNA was scheduled at least 4 weeks after the index FNA to reduce reactive atypia and to reflect routine clinical practice; procedures performed within 28 days were retained only when documented as clinically necessary, for example because of an inadequate initial sample or urgent clinical concern. For repeat FNA, we recorded the time interval from index FNA, the repeat cytology category, and subsequent downstream actions. For surgical management, we recorded surgery type (lobectomy or total thyroidectomy), timing relative to index FNA, and final histopathology of the index nodule. The interval from index FNA to repeat FNA was summarized as a continuous variable (median, interquartile range) and was explored in sensitivity analyses. The timing of repeat FNA was guided by institutional practice patterns informed by widely accepted international guidelines for thyroid nodule management, while allowing flexibility at the discretion of the attending physician based on ultrasound features, cytologic uncertainty, and overall clinical context. Variability in the interval to repeat FNA reflects real-world clinical decision-making, in which the timing is individualized according to the estimated malignancy risk, nodule characteristics, and patient preference rather than being fixed to a single predefined interval. Importantly, although repeat FNA was permitted after a minimum interval of 4 weeks, the observed median interval of 64 days indicates that most procedures were performed beyond the period of acute reactive cytologic changes and within a time frame generally considered appropriate in routine clinical practice for repeat cytologic assessment, thereby reducing the likelihood of poor sampling associated with excessively early rebiopsy.

### Outcomes and reference standard

The primary outcome was malignancy of the index nodule. When surgery was performed, the reference standard was final surgical histopathology. Malignancy included papillary thyroid carcinoma, follicular thyroid carcinoma, medullary thyroid carcinoma, poorly differentiated carcinoma, anaplastic carcinoma, and other malignant entities identified on histopathology. Because classification of noninvasive follicular thyroid neoplasm with papillary-like nuclear features (NIFTP) can influence implied risk estimates, the primary analysis treated NIFTP as non-malignant, with a prespecified sensitivity analysis classifying NIFTP as malignant.

For nodules without surgery, a hierarchical composite reference standard was applied. Nodules were classified as benign if they met at least one of the following criteria: (1) benign cytology (Bethesda II) on repeat FNA without subsequent evidence of malignancy; or (2) stable ultrasound appearance on serial follow-up for at least 12 months, defined as no development of new suspicious features and no clinically significant growth, defined as an increase in maximal diameter ≥20% with a minimum increase of 2 mm. Nodules were classified as malignant without surgery only if malignancy was confirmed by cytology (Bethesda VI) or if metastatic disease attributable to a thyroid primary was documented. The choice of a minimum 12-month stability criterion was prespecified and reflects commonly used clinical follow-up thresholds for low-risk thyroid nodules in routine care. We acknowledge the possibility of verification bias because surgical histopathology was not available for all nodules; therefore, we performed sensitivity analyses restricting the cohort to surgically confirmed nodules and, separately, to non-operated nodules with longer imaging follow-up of at least 24 months. As an additional robustness check, the benign-by-stability definition was repeated using an 18-month minimum follow-up threshold.

Secondary outcomes were: (1) unnecessary surgery, defined as diagnostic surgery performed after an index Bethesda III or IV result that yielded a non-malignant final diagnosis for the index nodule (including benign disease and NIFTP in the primary definition); and (2) diagnostic gain of repeat FNA, quantified as the proportion of nodules reclassified from Bethesda III/IV to an actionable cytology category after repeat FNA (Bethesda II, V, or VI) and the corresponding downstream change in management.

### Sample size considerations

The cohort size (N = 206) was fixed by the number of eligible Bethesda III/IV cases during the prespecified study period. Sample size adequacy was contextualized using three complementary criteria aligned with the study objectives. (1) Prediction model adequacy (events-per-parameter): with *k* predictor parameters, anticipated event proportion *p*, and a target events-per-parameter (EPP), the minimum sample size was approximated as:


$$N_{min}=\;\left(EPP\;\times k\right)/p.$$

We targeted EPP = 15 and assumed *p* = 0.30; therefore, we limited the predictor set to clinically grounded variables and applied penalized regression with bootstrap validation to reduce overfitting. (2) Precision for key proportions (binomial framework): for a proportion π and a desired two-sided 95% confidence interval half-width *d*, the required sample size was approximated as:


$$N=Z_{(1-}{\alpha_{/}}_{2)}2^{\times }\;\pi \left(1-\pi \right)/d^{2},$$

where *Z_(1-α/2)_* = 1.96 for *α* = 0.05. (3) Paired comparison of strategies (supportive): when contrasting the observed real-world strategy with the optimized pathway on the same nodules, discordant recommendations were evaluated using McNemar’s test, with the required sample size approximated as:


$$N=(Z_{(1-}{\alpha_{/}}_{2)}+Z{{(}_{1-}\beta_{)})}^{2}\times \;(p_{10}+\;p_{01})/{\left(p_{10}-\;p_{01}\right)}^{2},$$

where p_10_ and p_01_ denote the discordant proportions. Given the fixed cohort size, strategy comparisons were interpreted as supportive and reported with effect sizes and confidence intervals.

### Statistical analysis

Continuous variables were summarized as mean (standard deviation) or median (interquartile range) depending on distribution, and categorical variables as counts (percentages). Between-group comparisons used the independent-samples t test or Mann–Whitney U test for continuous variables and the chi-square test or Fisher’s exact test for categorical variables. All tests were two-sided with a significance level of 0.05.

To address the post-FNA decision problem, we performed two complementary analyses. First, malignancy risk was estimated using multivariable logistic regression incorporating prespecified clinical and ultrasound predictors and the Bethesda subclass (III *vs* IV). To reduce overfitting, penalized regression (ridge or lasso selected by cross-validation) was used as the primary modeling approach, with conventional logistic regression as a sensitivity analysis when event counts permitted. Discrimination was quantified using the area under the receiver operating characteristic curve. Calibration was evaluated using calibration intercept and slope and the Brier score. Internal validation and optimism correction were performed using bootstrap resampling (1,000 resamples). Clinical utility across plausible decision thresholds was examined using decision curve analysis. Second, diagnostic gain of repeat FNA was evaluated by calculating reclassification rates to actionable cytology categories, the proportion proceeding to surgery after repeat FNA, and the distribution of final diagnoses among those managed with repeat FNA versus immediate surgery. Unnecessary surgery rates were compared across ultrasound-derived risk strata and model-predicted risk strata. Within the operated subset, logistic regression was used to assess factors associated with unnecessary surgery. All regression and model-based analyses were conducted using a complete-case approach, including only participants with non-missing values for all covariates required by the corresponding analysis. Accordingly, the effective sample size may vary across analyses. Missingness was primarily observed in laboratory measurements and selected ultrasound descriptors that were not consistently available across all patients. Detailed missing data patterns and comparisons between complete-case and incomplete-case subsets are provided in **Supplementary Tables 1**, **2**. Descriptive analyses used available data for each variable, whereas multivariable modeling was restricted to complete cases for the included predictors.

The optimized post-FNA management pathway was constructed by linking model-predicted malignancy probabilities to clinically actionable decision thresholds. Three management options were defined: active surveillance, repeat FNA, and immediate surgery. Based on the distribution of predicted risk and clinical interpretability, two prespecified probability thresholds were used to guide decision-making. Nodules with a predicted malignancy probability below 0.20 were assigned to active surveillance, those with probabilities between 0.20 and 0.50 were assigned to repeat FNA, and those with probabilities above 0.50 were assigned to immediate surgery. These thresholds were selected through a pragmatic, hybrid approach combining decision curve analysis and clinical interpretability. Specifically, decision curve analysis was first used to identify a range of threshold probabilities associated with favorable net clinical benefit. Within this range, clinically meaningful cut-points were then chosen to reflect established management principles for indeterminate thyroid nodules, including conservative management at low risk, further diagnostic clarification at intermediate risk, and surgical intervention at higher risk. The final thresholds of 0.20 and 0.50 were selected to balance statistical performance with clinical applicability and ease of implementation, and were prespecified before pathway evaluation to avoid circular optimization. In practical terms, the optimized pathway functions as a rule-based algorithm in which each nodule is assigned a management recommendation solely based on its predicted probability of malignancy relative to these predefined thresholds. The optimized pathway was then applied to each nodule in the cohort, and resulting management recommendations were compared with observed clinical practice using paired analyses. All analyses were performed in R, version 4.3.2 (R Foundation for Statistical Computing, Vienna, Austria).

## Results

### Study population

**Figure 1:**
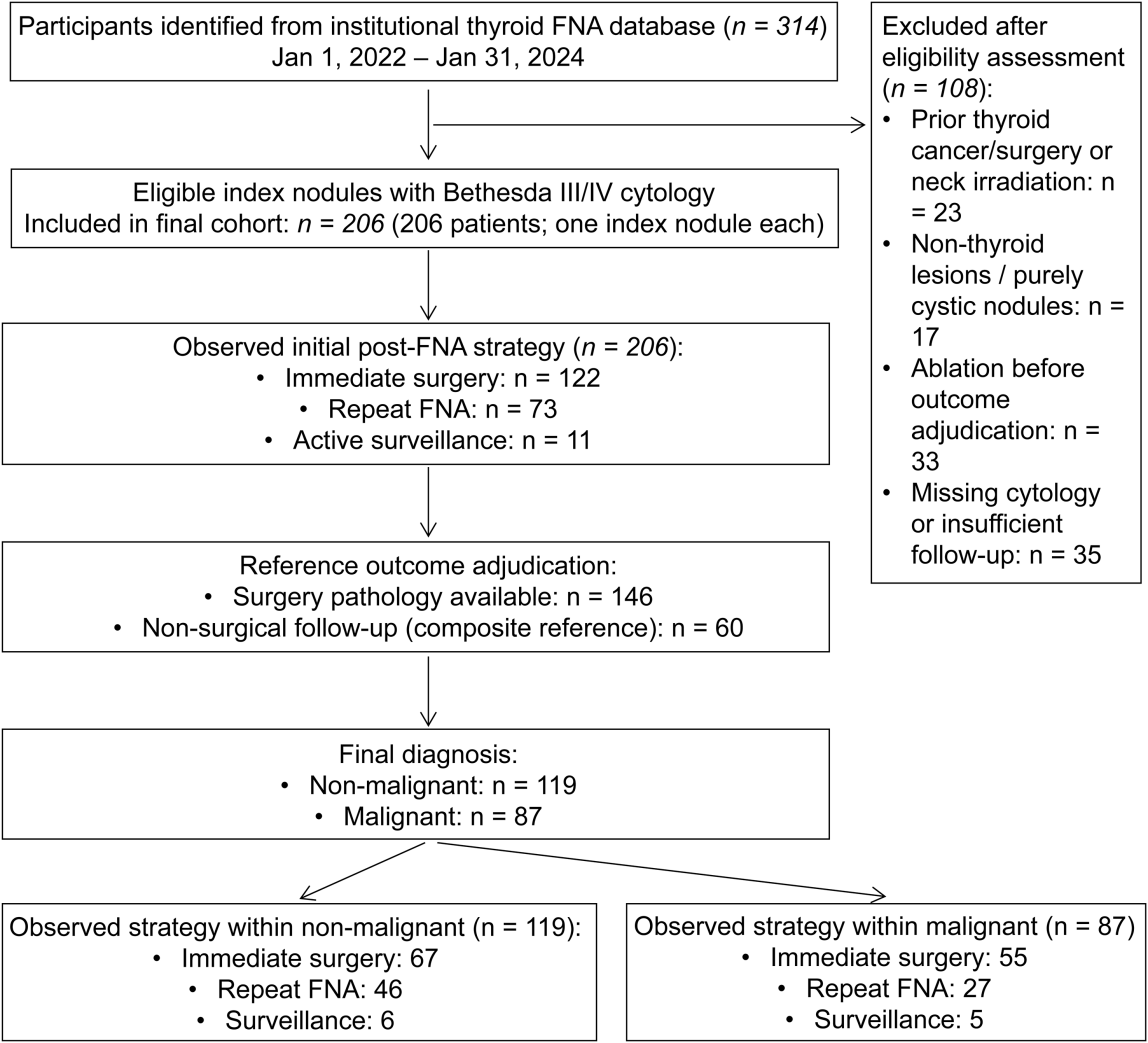
Study flowchart and cohort derivation. Flowchart illustrating patient selection, inclusion and exclusion criteria, and final cohort composition. A total of 314 patients with Bethesda III/IV thyroid nodules who underwent FNA, fine-needle aspiration were initially identified during the study period. After applying predefined inclusion and exclusion criteria, 206 patients were included in the final analytic cohort. Reasons for exclusion (n = 108) are detailed, including insufficient follow-up, missing key clinical or ultrasound data, non-diagnostic reference standards, and protocol deviations. The final cohort was used for subsequent clinical, ultrasound, and outcome analyses.

A total of 314 patients with Bethesda III or IV thyroid nodules were initially identified during the study period. After applying predefined inclusion and exclusion criteria, 206 patients with 206 index nodules were included in the final analytic cohort (**Figure 1**). Malignancy was confirmed in 87 nodules (42.2%), while 119 nodules (57.8%) were classified as non-malignant based on surgical histopathology or the prespecified composite reference standard.

Baseline clinical, biochemical, and ultrasound characteristics stratified by final diagnosis are summarized in **Table 1**. Demographic variables, including age, sex, and body mass index, were comparable between malignant and non-malignant groups. Most comorbid conditions and general clinical characteristics did not differ materially by outcome. In contrast, several ultrasound-derived features and composite ultrasound risk classifications were more frequently observed in malignant nodules.

**Table 1:** Baseline characteristics stratified by final diagnosis of malignancy.

Characteristic	Non-malignant (N = 119)	Malignant (N = 87)	*t*/*χ^2^*	*P* value
Age, years	48.68 ± 9.82	48.41 ± 11.44	-0.175	0.861
BMI, kg/m^2^	23.88 ± 2.79	23.94 ± 2.47	0.168	0.867
Max diameter, mm	18.55 ± 4.58	18.37 ± 3.78	-0.311	0.756
Volume, mL	3.73 ± 2.60	3.53 ± 2.31	-0.569	0.570
TSH, mIU/L	1.93 ± 0.43	1.82 ± 0.44	-1.561	0.120
FT4, pmol/L	15.45 ± 1.93	14.59 ± 1.57	-3.119	0.002
TPOAb, IU/mL	47.01 ± 13.93	36.29 ± 9.76	-2.753	0.007
TgAb, IU/mL	29.29 ± 10.64	27.90 ± 6.64	-0.412	0.681
HbA1c, %	5.62 ± 0.43	5.47 ± 0.40	-2.107	0.037
LDL-C, mmol/L	3.06 ± 0.66	2.87 ± 0.53	-1.956	0.052
ACR TI-RADS points	4.55 ± 1.93	5.79 ± 2.38	3.989	<0.001
Female sex	96 (80.7%)	63 (72.4%)	1.506	0.220
Current smoking	17 (14.3%)	6 (6.9%)	2.072	0.150
Hypertension	31 (26.1%)	22 (25.3%)	0.000	1.000
Diabetes	10 (8.4%)	12 (13.8%)	1.018	0.313
Dyslipidemia	26 (21.8%)	21 (24.1%)	0.048	0.827
Family history of thyroid cancer	6 (5.0%)	3 (3.4%)	0.043	0.835
Radiation exposure history	5 (4.2%)	3 (3.4%)	0.000	1.000
Autoimmune thyroiditis context	24 (20.2%)	17 (19.5%)	0.000	1.000
Symptomatic or palpable nodule	33 (27.7%)	21 (24.1%)	0.175	0.675
Multinodular goiter	37 (31.1%)	29 (33.3%)	0.036	0.850
Taller-than-wide shape	9 (7.6%)	19 (21.8%)	7.548	0.006
Suspicious cervical lymph nodes	7 (5.9%)	11 (12.6%)	2.096	0.148
Halo sign present	29 (24.4%)	26 (29.9%)	0.525	0.469
ACR TI-RADS TR5	20 (16.8%)	36 (41.4%)	14.114	<0.001
ATA high-suspicion pattern	57 (47.9%)	60 (69.0%)	8.251	0.004
Bethesda IV (*vs* III)	43 (36.1%)	40 (46.0%)	1.635	0.201
Observed: Repeat FNA	46 (38.7%)	27 (31.0%)	0.964	0.326
Observed: Surgery performed	79 (66.4%)	67 (77.0%)	2.258	0.133
Nodule location			0.573	0.751
Isthmus	4 (3.4%)	3 (3.4%)		
Left lobe	61 (51.3%)	40 (46.0%)		
Right lobe	54 (45.4%)	44 (50.6%)		
Composition			1.048	0.592
Mixed cystic-solid	34 (28.6%)	21 (24.1%)		
Solid/almost solid	82 (68.9%)	62 (71.3%)		
Spongiform	3 (2.5%)	4 (4.6%)		
Echogenicity			4.309	0.116
Hypoechoic	55 (46.2%)	41 (47.1%)		
Iso/hyperechoic	56 (47.1%)	33 (37.9%)		
Markedly hypoechoic	8 (6.7%)	13 (14.9%)		
Margins			1.605	0.658
Ill-defined	17 (14.3%)	13 (14.9%)		
Lobulated/irregular	28 (23.5%)	26 (29.9%)		
Smooth	70 (58.8%)	44 (50.6%)		
Suspected ETE	4 (3.4%)	4 (4.6%)		
Echogenic foci			6.856	0.077
Macrocalcifications	25 (21.0%)	11 (12.6%)		
None	60 (50.4%)	37 (42.5%)		
Punctate echogenic foci	22 (18.5%)	28 (32.2%)		
Rim calcifications	12 (10.1%)	11 (12.6%)		
Vascularity			5.728	0.126
Intranodular	31 (26.1%)	16 (18.4%)		
Mixed	27 (22.7%)	31 (35.6%)		
None/low	28 (23.5%)	14 (16.1%)		
Peripheral	33 (27.7%)	26 (29.9%)		
Elastography category			1.633	0.442
Intermediate	49 (41.2%)	30 (34.5%)		
Soft	43 (36.1%)	39 (44.8%)		
Stiff	27 (22.7%)	18 (20.7%)		
ACR TI-RADS category			15.460	0.001
TR2	7 (5.9%)	3 (3.4%)		
TR3	12 (10.1%)	7 (8.0%)		
TR4	80 (67.2%)	41 (47.1%)		
TR5	20 (16.8%)	36 (41.4%)		
ATA pattern			9.091	0.011
High suspicion	57 (47.9%)	60 (69.0%)		
Intermediate suspicion	53 (44.5%)	23 (26.4%)		
Low suspicion	9 (7.6%)	4 (4.6%)		
Observed initial strategy			1.276	0.528
Active surveillance	6 (5.0%)	5 (5.7%)		
Immediate surgery	67 (56.3%)	55 (63.2%)		
Repeat FNA	46 (38.7%)	27 (31.0%)		

Continuous variables are summarized as mean ± SD and categorical variables as n (%). For descriptive comparisons, denominators reflect available data for each variable. Groups were compared using the independent-samples *t* test for continuous variables and the chi-square test for categorical variables. Analyses were performed on available data for each variable, and two-sided *P*< 0.05 indicated statistical significance. BMI, body mass index; TSH, thyroid-stimulating hormone; FT4, free thyroxine; TPOAb, thyroid peroxidase antibody; TgAb, thyroglobulin antibody; HbA1c, glycated hemoglobin; LDL-C, low-density lipoprotein cholesterol; ACR, American College of Radiology; TI-RADS, Thyroid Imaging Reporting and Data System; TR, TI-RADS category; ATA, American Thyroid Association; FNA, fine-needle aspiration; ETE, extrathyroidal extension.

### Clinical and ultrasound characteristics associated with malignancy

Comparisons of clinical, biochemical, and ultrasound features between malignant and non-malignant nodules are presented in **Table 1**. Among laboratory parameters, lower free thyroxine (FT4) levels were more frequently observed in malignant nodules. Nodule size and location did not differ significantly between groups.

Several individual ultrasound features showed between-group differences. Malignant nodules more commonly exhibited taller-than-wide shape, marked hypoechogenicity, punctate echogenic foci, and suspicious cervical lymph nodes. High-risk ultrasound classifications, including ACR TI-RADS TR5 and ATA high-suspicion patterns, were also more prevalent among malignant nodules.

### Univariable and multivariable analyses of predictors of malignancy

Results of univariable and multivariable logistic regression analyses are shown in **Table 2**. In univariable analyses, multiple ultrasound features and composite ultrasound risk scores were associated with malignancy. Lower FT4 levels and higher HbA1c values were also associated with malignant outcomes on univariable testing.

**Table 2:** Clinical and ultrasound predictors of malignancy on the reference standard.

Predictor	Univ OR	Univ 95% CI	Univ P	Multiv OR	Multiv 95% CI	Multiv P
Age (per 10 years)	0.98	0.75–1.27	0.857	—	—	—
Male sex	1.59	0.83–3.06	0.165	—	—	—
BMI (kg/m^2^)	1.01	0.91–1.12	0.868	—	—	—
Current smoking	0.44	0.17–1.18	0.103	—	—	—
Hypertension	0.96	0.51–1.81	0.902	—	—	—
Diabetes	1.74	0.72–4.24	0.220	—	—	—
Dyslipidemia	1.14	0.59–2.19	0.699	—	—	—
Family history of thyroid cancer	0.67	0.16–2.77	0.583	—	—	—
Radiation exposure history	0.81	0.19–3.50	0.783	—	—	—
Autoimmune thyroiditis context	0.96	0.48–1.92	0.911	—	—	—
Symptomatic or palpable nodule	0.83	0.44–1.56	0.563	—	—	—
Multinodular goiter	1.11	0.61–2.00	0.734	—	—	—
TSH (mIU/L)	0.57	0.28–1.16	0.121	—	—	—
FT4 (pmol/L)	0.76	0.63–0.91	0.004	0.63	0.48–0.84	0.001
TPOAb positivity (≥34 IU/mL)	0.64	0.31–1.32	0.228	—	—	—
TgAb positivity (≥40 IU/mL)	0.71	0.30–1.67	0.427	—	—	—
HbA1c (%)	0.43	0.19–0.97	0.042	—	—	—
LDL-C (mmol/L)	0.60	0.35–1.03	0.064	—	—	—
Max diameter (per 10 mm)	0.90	0.47–1.74	0.762	—	—	—
Solid or almost solid composition	1.12	0.61–2.05	0.716	—	—	—
Taller-than-wide shape	3.42	1.46–7.98	0.005	3.69	1.21–11.27	0.021
Irregular margins or suspected ETE	1.43	0.79–2.61	0.242	—	—	—
Marked hypoechogenicity	2.44	0.96–6.17	0.060	3.12	1.01–9.64	0.048
Hypoechogenicity (non-marked)	1.04	0.60–1.81	0.897	—	—	—
Punctate echogenic foci	2.09	1.10–3.99	0.025	2.01	1.01–4.89	0.046
Macrocalcifications	0.54	0.25–1.18	0.122	—	—	—
Rim calcifications	1.29	0.54–3.08	0.565	—	—	—
Halo sign present	1.32	0.71–2.46	0.377	—	—	—
Suspicious cervical lymph nodes	2.32	0.86–6.24	0.097	4.31	1.10–16.90	0.036
Elastography stiffness	0.89	0.45–1.74	0.732	—	—	—
ACR TI-RADS points (per 5 points)	3.91	1.95–7.86	<0.001	—	—	—
ACR TI-RADS TR5 category	3.49	1.84–6.64	<0.001	—	—	—
ATA high-suspicion pattern	2.42	1.35–4.31	0.003	—	—	—
Bethesda IV (vs III)	1.50	0.86–2.64	0.156	2.14	0.89–5.14	0.089

Univariable models were fitted using all available data for each predictor. The multivariable model represents a prespecified final model fitted in complete cases (n = 121) and included a limited number of clinically relevant variables to avoid overfitting and multicollinearity. Composite ultrasound risk scores (ACR TI-RADS and ATA patterns) were not entered into the multivariable model together with their component sonographic features. OR, odds ratio; CI, confidence interval; BMI, body mass index; TSH, thyroid-stimulating hormone; FT4, free thyroxine; TPOAb, thyroid peroxidase antibody; TgAb, thyroglobulin antibody; HbA1c, glycated hemoglobin; LDL-C, low-density lipoprotein cholesterol; ETE, extrathyroidal extension; ACR, American College of Radiology; TI-RADS, Thyroid Imaging Reporting and Data System; TR, TI-RADS category; ATA, American Thyroid Association; AUC, area under the receiver operating characteristic curve; Brier score, mean squared error of probabilistic predictions.

**Figure 2:**
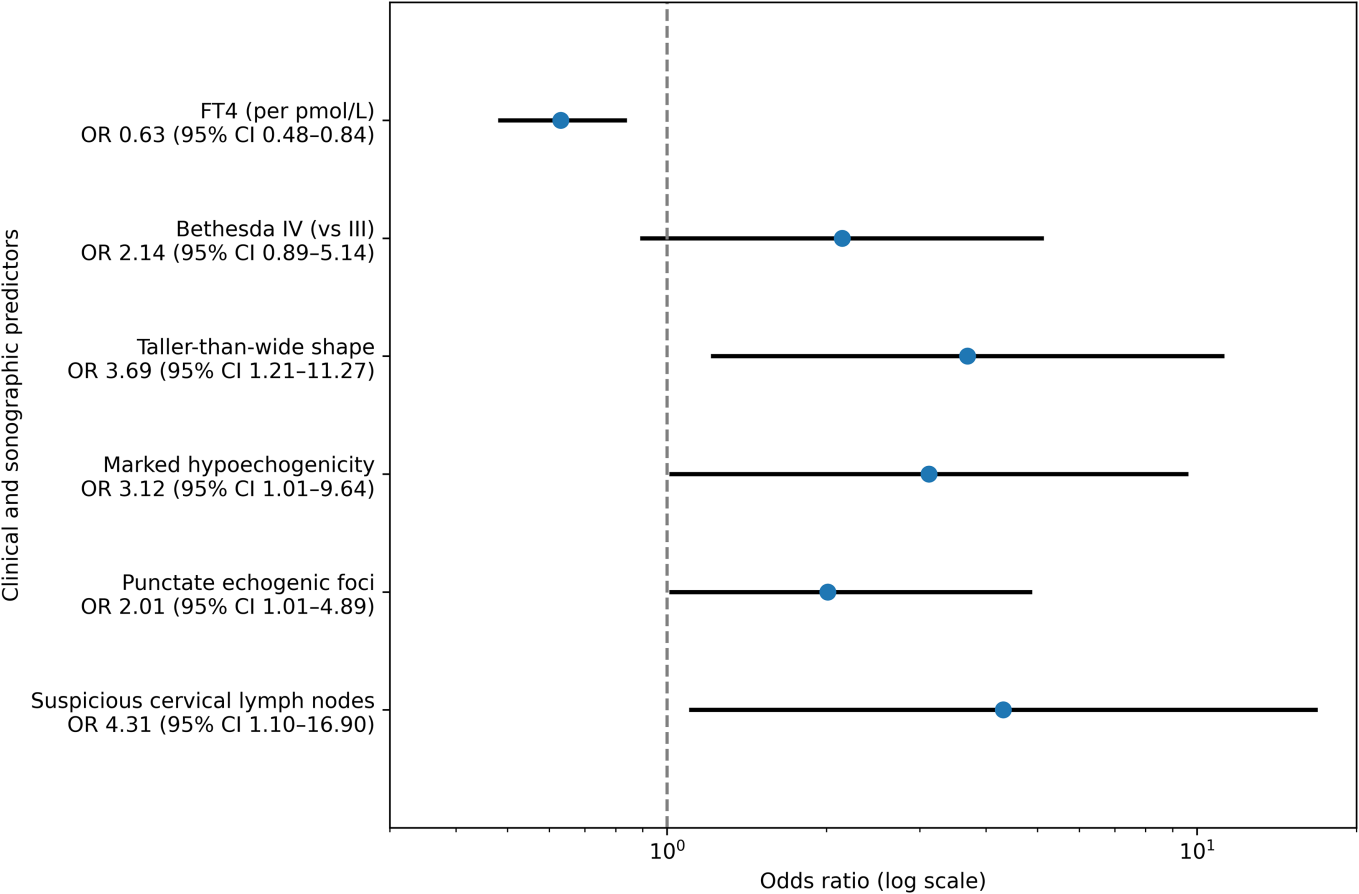
Independent predictors of malignancy in the multivariable model. Forest plot displaying adjusted ORs, odds ratios with 95% Cis, confidence intervals for independent predictors of malignancy identified in the multivariable logistic regression model. Clinical and ultrasound variables retained in the final model are shown along the y-axis, with corresponding effect estimates plotted on a logarithmic scale. Values of OR and 95% CI are displayed beneath each predictor label to facilitate interpretation.

The multivariable model was fitted in complete cases (n = 121) using prespecified clinically relevant predictors. The reduction in sample size was mainly attributable to missing values in laboratory variables and selected ultrasound features required for model construction. No meaningful differences in baseline characteristics or outcome distribution were observed between complete-case and incomplete-case subsets (**Supplementary Table 2**). After adjustment, lower FT4 levels remained independently associated with malignancy. Among ultrasound features, taller-than-wide shape, marked hypoechogenicity, punctate echogenic foci, and suspicious cervical lymph nodes were retained as independent predictors. Bethesda IV cytology demonstrated a borderline association after adjustment. Composite ultrasound scores were intentionally excluded from the multivariable model to avoid collinearity with their component features. Adjusted effect estimates for variables retained in the final multivariable model are visualized in **Figure 2**.

### Model discrimination and risk stratification performance

**Figure 3:**
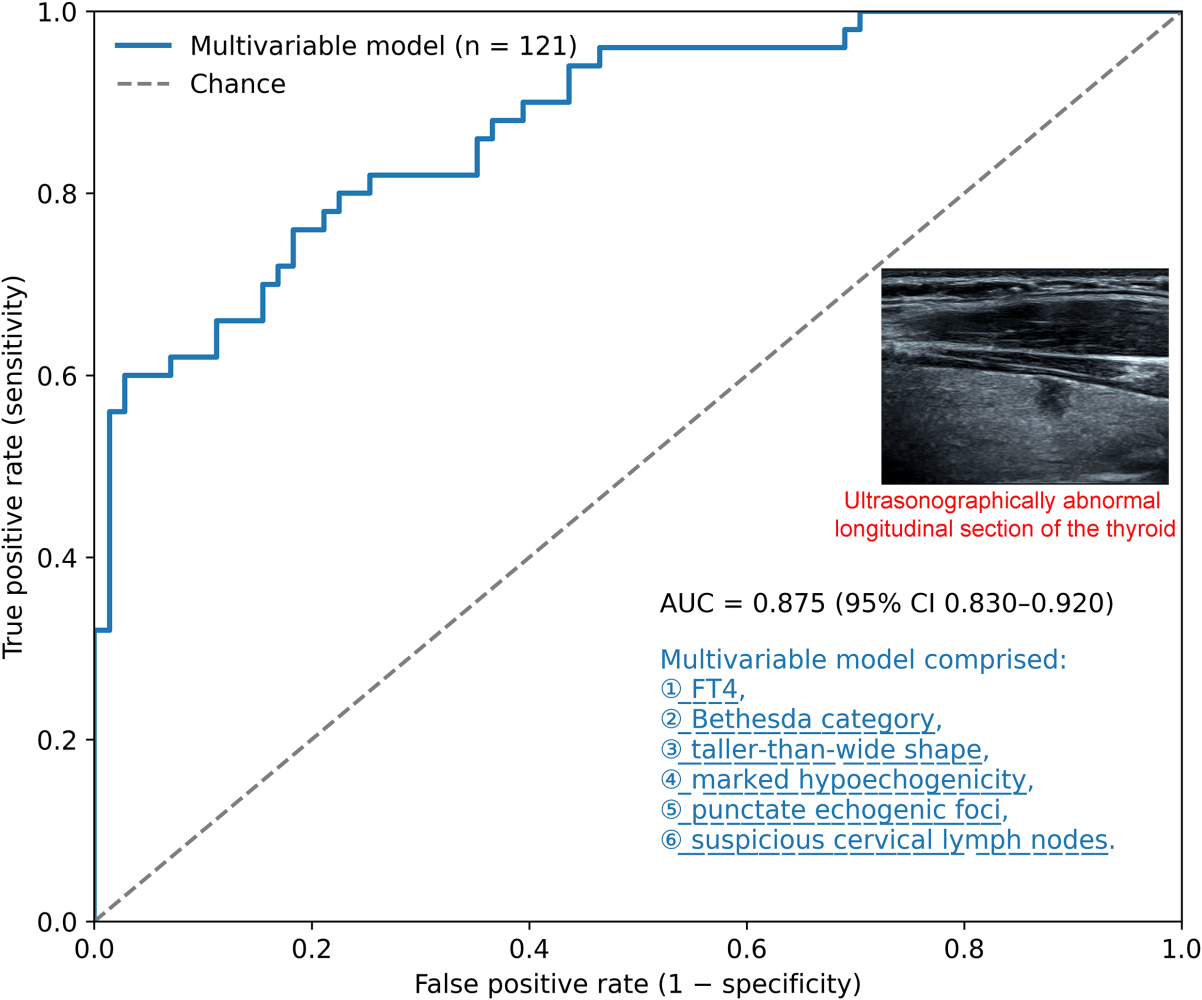
Discrimination performance of the multivariable prediction model. ROC, receiver operating characteristic curve of the multivariable model for predicting malignancy in Bethesda III and IV thyroid nodules. The model demonstrated good discriminative performance, with an apparent AUC of 0.875 and an optimism-corrected AUC of 0.798 obtained from bootstrap internal validation. The multivariable model incorporated six clinically and sonographically relevant predictors: FT4, free thyroxine, Bethesda category, taller-than-wide nodule shape, marked hypoechogenicity, punctate echogenic foci, and suspicious cervical lymph nodes. The dashed diagonal line represents chance-level discrimination.

The discriminative performance of the multivariable model is illustrated in **Figure 3**. The model demonstrated an apparent AUC of 0.875 (**Figure 3**), with an optimism-corrected AUC of 0.798 obtained from bootstrap validation. The receiver operating characteristic curve showed clear separation from the reference line, indicating good discrimination for malignancy among Bethesda III and IV nodules. Model performance metrics, including the area under the curve, Brier score, and calibration slope, are summarized in **Table 2**, indicating acceptable overall accuracy and calibration.

**Figure 4:**
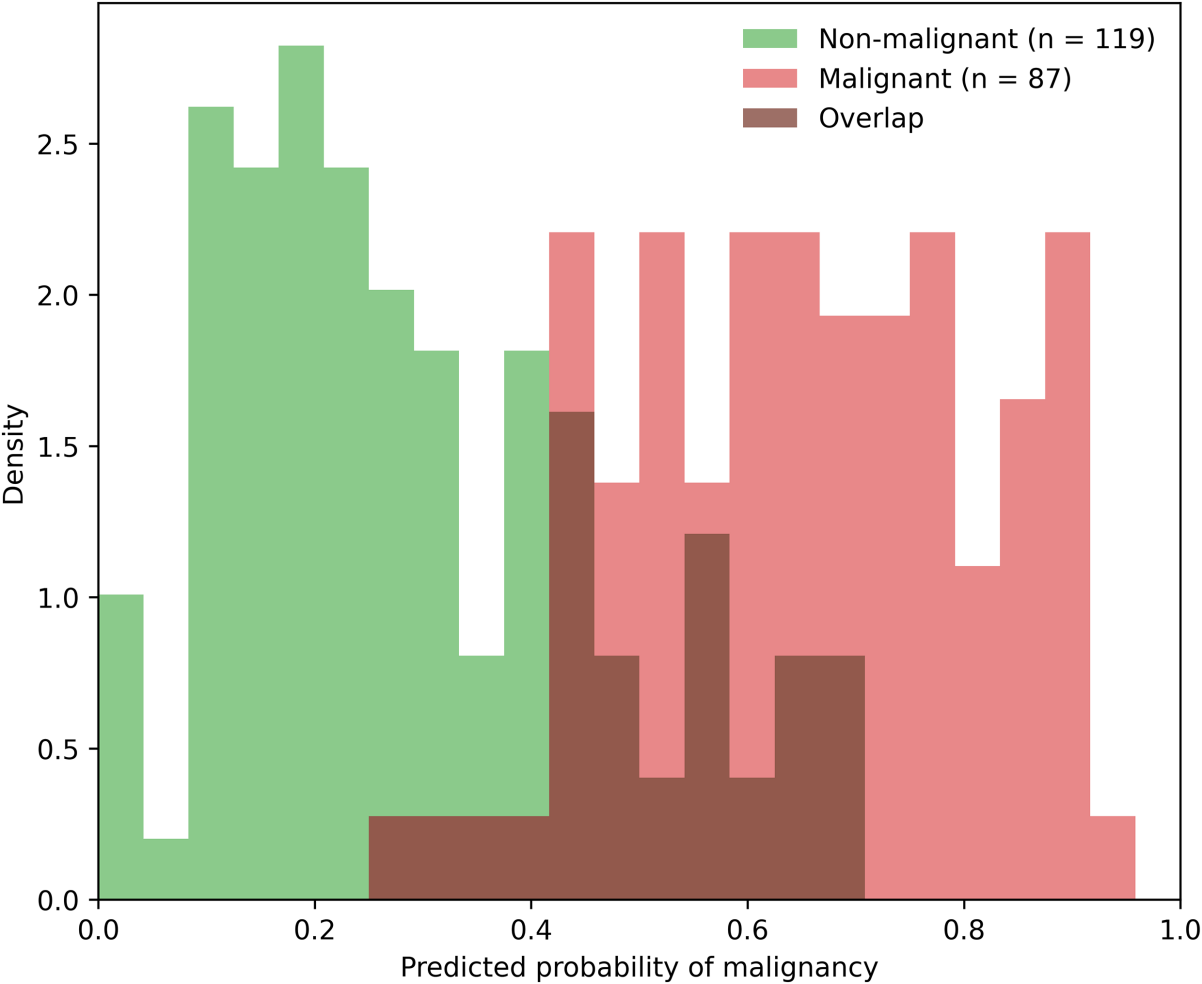
Distribution of predicted malignancy risk by final diagnosis. Distribution of model-predicted malignancy risk stratified by final diagnostic outcome. Density curves illustrate predicted risk among non-malignant nodules, malignant nodules, and their overlapping distribution. The figure demonstrates partial separation between diagnostic groups while highlighting areas of risk overlap, reflecting the inherent uncertainty in individualized post-FNA risk assessment.

The distribution of predicted malignancy risk stratified by final diagnostic outcome is shown in **Figure 4**. Predicted risk values tended to be higher among malignant nodules, although substantial overlap between malignant and non-malignant groups was observed. Although the model demonstrated good discriminative performance, predicted risk estimates were not perfectly concordant with surgical pathology outcomes, reflecting the inherent uncertainty in risk prediction for indeterminate thyroid nodules.

### Diagnostic yield of repeat FNA

Repeat FNA was performed in 73 patients (35.4%), at a median interval of 64 days from the index biopsy (**Table 3**). More than half of repeat FNAs resulted in cytologic reclassification to a non-indeterminate category. Upgrading to Bethesda V or VI occurred in a subset of cases and was more frequently observed among nodules initially classified as Bethesda III.

**Table 3:** Diagnostic yield of repeat fine-needle aspiration and cytology reclassification.

Item	Value	Notes
Repeat FNA analysis		
Repeat FNA performed, n (%)	73 (35.4%)	
Interval to repeat FNA, days (median [IQR])	64 [53, 81]	
Actionable reclassification (II/V/VI), n (%)	39 (53.4%)	
Upgraded to Bethesda V/VI, n (%)	7 (9.6%)	
Repeat cytology distribution		
Category	n (%)	
II	32 (43.8%)	
III	22 (30.1%)	
IV	12 (16.4%)	
V	6 (8.2%)	
VI	1 (1.4%)	
Actionable reclassification by Bethesda subgroup		
Stratum	Actionable reclassification	Upgraded to V/VI
Bethesda III	31 (60.8%)	6 (11.8%)
Bethesda IV	8 (36.4%)	1 (4.5%)

The interval from index fine-needle aspiration to repeat fine-needle aspiration is summarized as median with interquartile range. Cytology outcomes after repeat sampling are presented as n with percentage, including actionable categories Bethesda II, V, and VI. Proportions were compared using the chi-square test when applicable. Two-sided *P*< 0.05 indicated statistical significance. FNA, fine-needle aspiration; IQR, interquartile range.

A proportion of nodules remained indeterminate following repeat sampling, indicating variable diagnostic yield of rebiopsy across the cohort.

### Timing of post-FNA management strategies

**Figure 5:**
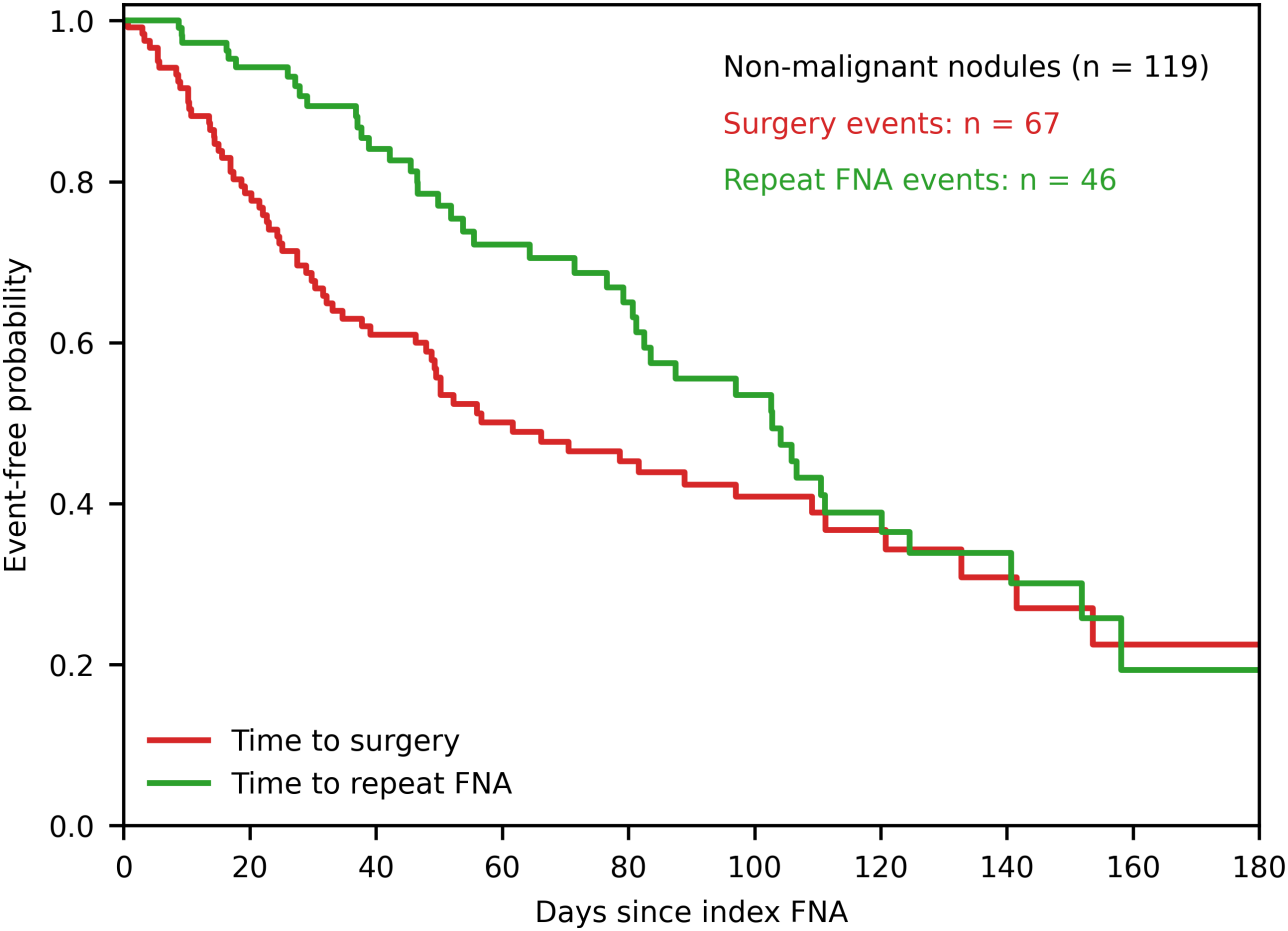
Timing of post-FNA management strategies in non-malignant nodules. Kaplan–Meier curves depicting time to definitive surgery and time to repeat FNA, fine-needle aspiration among patients with non-malignant thyroid nodules. The x-axis represents days since the index FNA, and the y-axis represents event-free probability. Curves illustrate the temporal distribution of post-FNA management decisions, highlighting differences in the timing of surgical intervention versus repeat cytologic assessment during follow-up.

Time-to-event analyses for post-FNA management decisions among non-malignant nodules are shown in **Figure 5**. Surgical intervention tended to occur earlier following the index FNA, whereas repeat FNA was distributed more evenly over follow-up. These temporal patterns reflect differences in clinical management pathways after initial cytologic assessment.

### Comparison of observed management and the optimized pathway

**Figure 6:**
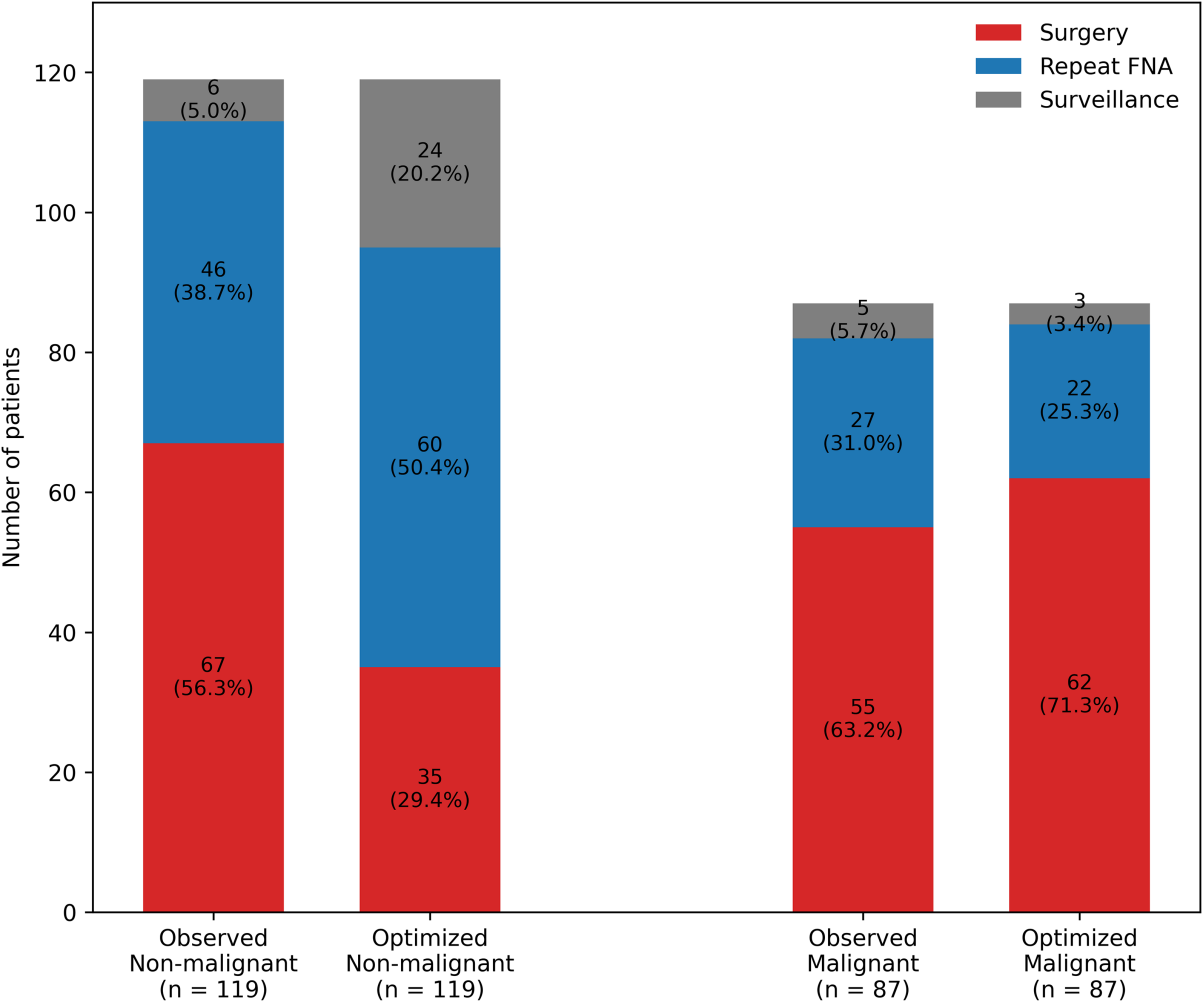
Reclassification of post-FNA management decisions using the optimized pathway. Reclassification of post-FNA management strategies under the optimized pathway compared with observed clinical practice. Stacked bar plots depict the proportion and absolute number of patients reassigned to surgery or surveillance based on optimized risk stratification. Percentages and counts are shown within each bar to illustrate shifts in clinical decision-making and potential reduction of unnecessary interventions.

The optimized pathway applied prespecified risk thresholds (<0.20 for surveillance, 0.20–0.50 for repeat FNA, and >0.50 for surgery) to guide management decisions. Observed post-FNA management decisions and those recommended by the optimized post-FNA pathway are compared in **Table 4**; **Figure 6**. Unnecessary surgery was defined as surgery yielding benign pathology or NIFTP. When restricted to operated nodules (n = 146), the corresponding rate of unnecessary surgery was 54.1%, indicating that more than half of surgical procedures resulted in non-malignant pathology. Application of the optimized pathway resulted in a lower proportion of nodules assigned to immediate surgery compared with observed clinical practice. The frequency of repeat FNA was similar between strategies.

**Table 4:** Paired comparison of observed management and the optimized pathway.

Outcome metric	Observed strategy	Optimized pathway	Absolute difference (Obs−Opt)	Paired test (McNemar) *P*
Surgery recommended/performed, n (%)	146 (70.9%)	119 (57.8%)	13.1%	0.005
Unnecessary surgery among all nodules (benign or NIFTP), n (%)	79 (38.3%)	60 (29.1%)	9.2%	
Repeat FNA, n (%)	73 (35.4%)	74 (35.9%)	-0.5%	

Observed management and optimized recommendations were compared within the same nodules. Paired differences in surgery recommendation were evaluated using McNemar’s test. “Unnecessary surgery among all nodules” was calculated using the full cohort (N = 206) as the denominator. Results are reported as n with percentage and absolute differences. Two-sided *P*< 0.05 indicated statistical significance. FNA, fine-needle aspiration; NIFTP, noninvasive follicular thyroid neoplasm with papillary-like nuclear features; Obs, observed strategy; Opt, optimized pathway.

**Figure 7:**
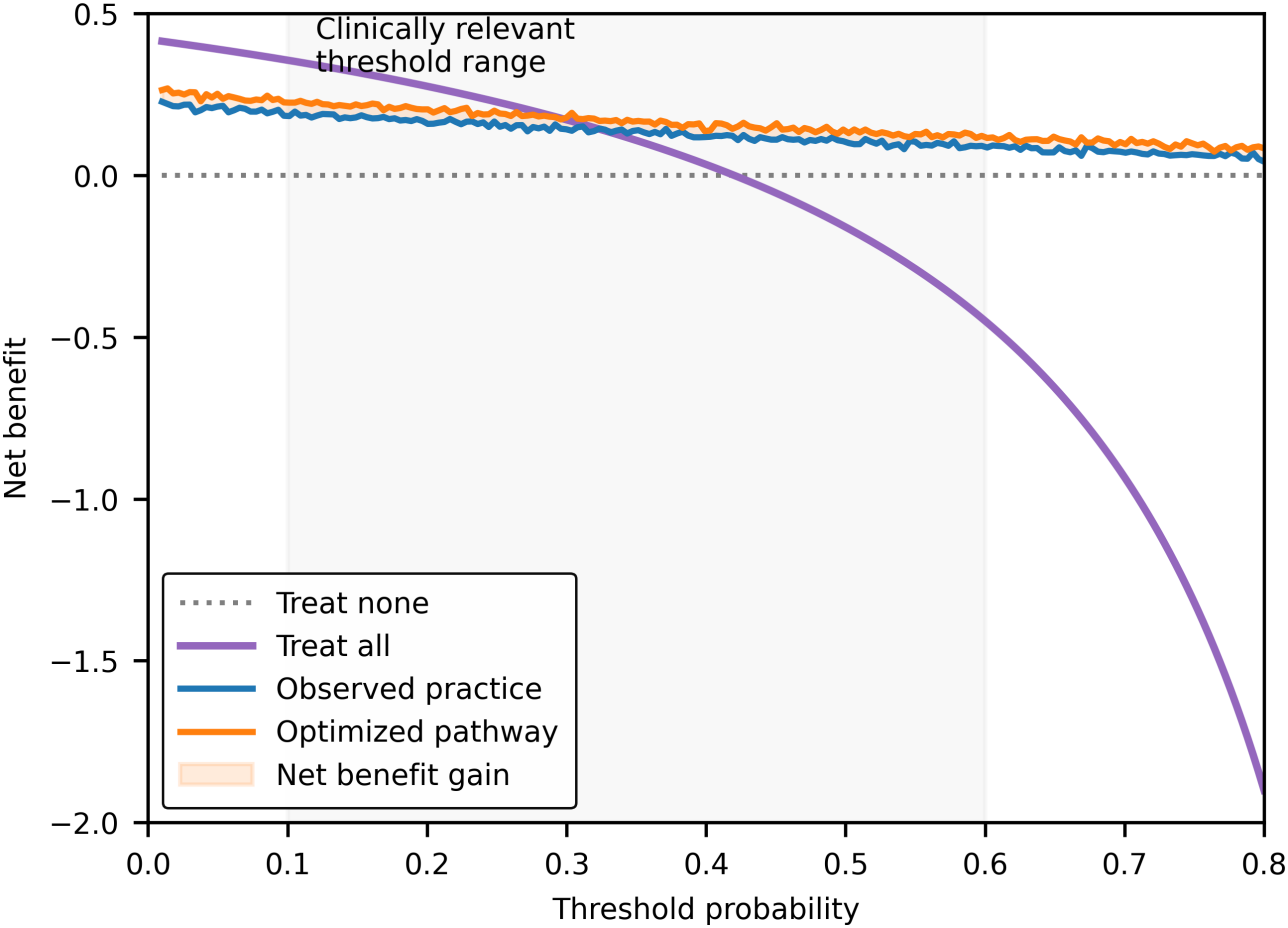
Clinical utility of the optimized post-FNA management pathway. Decision curve analysis comparing the net clinical benefit of the optimized post-FNA management pathway with observed clinical practice across a range of threshold probabilities. The optimized pathway consistently demonstrates greater net benefit than observed practice, as well as treat-all and treat-none strategies, within clinically relevant threshold probability ranges. Shaded areas indicate threshold regions in which the optimized pathway yields incremental net benefit over observed practice.

Decision curve analysis demonstrated that the optimized pathway provided greater net clinical benefit than observed practice across a broad range of clinically relevant threshold probabilities (**Figure 7**). The optimized strategy also showed higher net benefit than treat-all and treat-none approaches.

### Sensitivity analyses

Sensitivity analyses are summarized in **Table 5**. When analyses were restricted to surgically confirmed nodules, malignancy prevalence increased as expected, while estimates of unnecessary surgery remained consistent with the primary analysis. Among nodules managed non-surgically with extended follow-up, malignancy rates were lower and repeat FNA was frequently performed. Across all sensitivity analyses, the direction and magnitude of the main findings were stable. In the prespecified sensitivity analysis in which noninvasive follicular thyroid neoplasm with papillary-like nuclear features was classified as malignant, malignancy prevalence increased from 42.2% to 44.7%, while the proportion of unnecessary surgery among operated nodules decreased from 54.1% to 50.7%, with no material change in the relative performance of the optimized pathway.

**Table 5:** Prespecified sensitivity analyses across alternative verification and follow-up subsets.

Subset	N	Malignancy (primary)	Surgery rate	Unnecessary surgery among surgeries	Repeat FNA rate
Primary analysis (full cohort)	206	42.2%	70.9%	54.1%	35.4%
NIFTP classified as malignant	206	44.7%	70.9%	50.7%	35.4%
Surgery pathology only	146	45.9%	100.0%	54.1%	15.1%
Non-surgery follow-up ≥24 months	33	30.3%	0.0%		84.8%
Non-surgery follow-up ≥18 months	46	34.8%	0.0%		84.8%

Sensitivity analyses included the surgery pathology-only subset, the non-surgery subset with follow-up of at least 24 months, and an alternative benign stability threshold using follow-up of at least 18 months. Unnecessary surgery among surgeries was calculated using the number of surgically treated nodules as the denominator. In the sensitivity analysis classifying noninvasive follicular thyroid neoplasm with papillary-like nuclear features as malignant, malignancy prevalence increased modestly, while the proportion of unnecessary surgery decreased accordingly. FNA, fine-needle aspiration; N, number of nodules.

To further assess measurement consistency, we compared ultrasound-derived maximal diameter with pathological size in the surgically treated subset (n = 146). The mean maximal diameter measured on ultrasound was 18.47 ± 4.29 mm, compared with 17.92 ± 4.11 mm on surgical pathology. The two measurements showed good agreement, with no significant systematic difference (paired *t*-test, *P* = 0.214), and a strong positive linear correlation between the two measurements (Pearson correlation coefficient *r* = 0.82, *P*< 0.001). These findings suggest that ultrasound-based size assessment was broadly consistent with pathological measurements and is unlikely to have introduced substantial bias into the predictive modeling.

## Discussion

Clinical decision-making after an indeterminate thyroid FNA remains one of the most persistent challenges in contemporary thyroid practice (17). Bethesda III and IV nodules occupy a diagnostically ambiguous space in which the competing risks of missed malignancy and unnecessary surgery must be carefully balanced (18). The observed malignancy prevalence in the present cohort was higher than the commonly cited Bethesda reference ranges. This finding is most likely explained by selection effects inherent to retrospective studies of post-FNA management, in which nodules referred for surgery tend to represent a higher-risk subset. In addition, institutional case mix and local referral patterns may further influence the observed distribution of malignancy. The classification of noninvasive follicular thyroid neoplasm with papillary-like nuclear features also contributes to variability in reported malignancy rates across studies. In our sensitivity analysis treating this entity as malignant, the overall malignancy prevalence increased modestly, while the relative performance of the optimized pathway remained stable. These observations highlight the importance of interpreting malignancy risk within the clinical context in which decisions are made, rather than relying solely on generalized Bethesda estimates. Within this setting, the present study proposes an optimized and clinically grounded post-FNA management pathway that integrates routinely available clinical and ultrasound features to support individualized risk stratification and downstream decision-making. Rather than introducing novel biomarkers or proprietary assays, the proposed approach emphasizes pragmatic optimization of existing information and aligns diagnostic intensity with estimated malignancy risk.

An unexpected finding of the present study was the independent association between lower free thyroxine levels and malignancy. This observation is not readily explained by established biological mechanisms and should be interpreted with caution. While prior studies have occasionally reported similar associations, the underlying pathophysiological basis remains unclear, and a direct causal relationship is not well supported. Several alternative explanations merit consideration. First, this finding may reflect residual confounding or case-mix effects inherent to retrospective analyses, particularly if patients with certain clinical profiles were more likely to undergo surgery or more intensive evaluation. Second, thyroid function testing in routine practice is subject to temporal variability and laboratory-related factors, which may introduce measurement heterogeneity across the study period. Third, the lack of a significant association with thyroid peroxidase antibody positivity in the present analysis makes an autoimmune-mediated explanation less straightforward. Taken together, these considerations suggest that the observed association between lower free thyroxine levels and malignancy may not represent a direct biological effect, but rather a context-dependent finding influenced by underlying clinical and methodological factors. In contrast, the ultrasound-based predictors identified in this study, including taller-than-wide shape, marked hypoechogenicity, punctate echogenic foci, and suspicious cervical lymph nodes, are broadly consistent with prior literature emphasizing the prognostic relevance of sonographic morphology in indeterminate nodules (19–21). Importantly, composite ultrasound risk classifications such as ACR TI-RADS and ATA high-suspicion patterns were not retained in the final model because their predictive information largely overlaps with that provided by individual ultrasound features. This modeling strategy prioritizes parsimony and interpretability, which are essential when prediction tools are intended to inform real-world clinical decision-making rather than purely statistical classification. In addition, the observed agreement between ultrasound-derived and pathological size measurements supports the reliability of ultrasound-based variables included in the predictive model.

Beyond discrimination performance, the present study advances the field by explicitly linking risk prediction to subsequent management decisions. Previous investigations of indeterminate thyroid nodules have frequently focused on diagnostic accuracy or malignancy prediction in isolation, without assessing how such information translates into differences in clinical management (22). In contrast, the current analysis integrates prediction modeling with time-to-decision analyses, reclassification of management strategies, and evaluation of clinical utility. The observed timing patterns of surgery and repeat FNA illustrate that post-FNA decisions follow distinct trajectories, reflecting heterogeneous thresholds for intervention in routine practice. When applied to these decision points, the optimized pathway reduced the proportion of nodules directed to immediate surgery while maintaining similar use of repeat biopsy. This finding is particularly relevant in light of growing concerns regarding overtreatment of indolent thyroid lesions, including noninvasive follicular thyroid neoplasm with papillary-like nuclear features, and aligns with broader international efforts to promote more conservative and risk-adapted management strategies.

An important observation in the present study is the differential diagnostic yield of repeat FNA between Bethesda III and Bethesda IV nodules. The rate of actionable cytologic reclassification was substantially higher in Bethesda III nodules compared with Bethesda IV nodules, indicating that repeat FNA provides greater diagnostic resolution in the former group. This finding is consistent with the known biological and cytological characteristics of these categories, as follicular-patterned lesions represented in Bethesda IV are less amenable to definitive classification on repeat cytology. From a clinical perspective, this difference has important implications for post-FNA management. The higher yield of repeat FNA in Bethesda III nodules supports its role as a key intermediate step in risk stratification, whereas its more limited incremental value in Bethesda IV nodules suggests that reliance on repeat cytology alone may be insufficient for decision-making in this subgroup. These observations are in line with recent evidence from large prospective studies, including the ELATION trial (23), which have highlighted the heterogeneous utility of repeat FNA across indeterminate cytology categories. Although the optimized pathway in the present study was constructed based on continuous risk estimates rather than cytologic category alone, these findings suggest that future refinements could incorporate Bethesda subclass–specific considerations to further improve clinical applicability and decision efficiency.

The clinical relevance of the optimized pathway is further supported by decision curve analysis. Across a wide range of clinically relevant threshold probabilities, the optimized strategy consistently demonstrated greater net benefit than observed practice as well as strategies that treat all or treat none (7, 15, 24). Decision curve analysis explicitly incorporates the relative harms associated with false-positive and false-negative decisions, which makes it particularly well suited for evaluating management approaches in diagnostically indeterminate conditions. The consistent net benefit observed across thresholds suggests that incremental improvements in individualized risk estimation can translate into meaningful gains in patient-centered outcomes, especially when applied across large patient populations. These findings add to a growing body of literature advocating for the integration of decision-analytic methods alongside traditional measures of diagnostic performance.

From a broader perspective, the present findings have implications for future refinement of post-FNA management algorithms. As molecular testing becomes increasingly accessible, there is rising interest in strategies that integrate molecular results with clinical and ultrasound features rather than relying on any single modality in isolation (25–28). The framework proposed in this study provides a foundation for such integration, allowing additional data layers to be incorporated in a modular and clinically interpretable manner. Future prospective studies could evaluate sequential or conditional testing strategies guided by baseline risk estimates, with the goal of further improving diagnostic efficiency and resource utilization. External validation in diverse practice settings will also be necessary to assess generalizability, particularly in regions with differing disease prevalence, ultrasound expertise, and thresholds for surgical intervention.

Several limitations of this study merit consideration. First, the retrospective design introduces the potential for selection bias and unmeasured confounding, particularly with respect to clinician-driven decisions regarding surgery or repeat FNA. Although prespecified criteria and sensitivity analyses were used to address these concerns, causal inference cannot be established. Second, the multivariable model was developed using a complete-case approach, which reduced the effective sample size and may have limited the precision of effect estimates. Missing data were primarily related to laboratory measurements and certain ultrasound descriptors that were not uniformly recorded in routine practice. Although comparison between complete-case and non-complete-case subsets did not suggest substantial systematic differences, the potential for bias cannot be entirely excluded. Alternative approaches, such as multiple imputation, should be considered in future studies. Third, the composite reference standard that incorporated both surgical pathology and extended clinical follow-up may have resulted in misclassification of a small number of indolent malignancies among nodules managed non-surgically. Nevertheless, this approach reflects real-world clinical practice and has been widely adopted in studies of indeterminate thyroid nodules. Fourth, the study was conducted at a single center, and local practice patterns, imaging expertise, and patient preferences may limit the generalizability of the findings. External validation in independent cohorts is therefore essential before broader implementation. Fifth, although the optimized pathway reduced unnecessary surgery at the population level, individual patient values and preferences were not explicitly modeled and remain critical determinants of management decisions in clinical care.

An additional and important consideration relates to the external validity and cross-context applicability of the proposed management pathway. The present study was conducted in a single tertiary center in China, and several key factors may differ from clinical practice environments in Europe and North America. First, the observed malignancy prevalence in Bethesda III and IV nodules in this cohort was relatively high, likely reflecting local referral patterns and surgical selection thresholds. In contrast, malignancy rates reported in Western cohorts are often lower, particularly in systems where more conservative surveillance strategies are routinely adopted. Second, thresholds for proceeding to diagnostic surgery may vary substantially across healthcare systems, influenced by differences in patient preference, medico-legal context, access to molecular testing, and institutional practice norms. Third, although ultrasound risk stratification systems such as ACR TI-RADS and ATA patterns are internationally recognized, their implementation and interpretative thresholds may differ in routine practice, and operator-dependent variability may further affect risk categorization. In this context, the present study applied the 2015 ATA framework; more recent updates have been proposed, which may further refine risk stratification and management strategies in contemporary practice. Importantly, the proposed post-FNA management pathway was developed within a framework primarily informed by locally observed practice patterns and may not be directly transferable to guideline-driven environments such as those structured by the American Thyroid Association, the British Thyroid Association, or the European Association of Nuclear Medicine. In these settings, post-FNA decision-making is often more explicitly protocolized and may incorporate additional elements, including molecular diagnostics or structured risk-based follow-up algorithms. Therefore, rather than being interpreted as a prescriptive or universally applicable algorithm, the optimized pathway presented in this study should be considered a flexible, data-informed framework that may require recalibration of risk thresholds and adaptation to local disease prevalence, resource availability, and guideline context. Future studies should focus on external validation of this model in diverse geographic and clinical settings, including direct comparisons within ATA- and BTA-guided management pathways. Prospective evaluation incorporating molecular testing and multi-center cohorts will be essential to determine the robustness, transportability, and clinical utility of the proposed strategy across different healthcare systems.

In summary, this study presents a pragmatic and evidence-based approach to optimizing post-FNA management of Bethesda III and IV thyroid nodules through integration of readily available clinical and ultrasound features into a unified decision framework. By extending analysis beyond diagnostic accuracy to include management timing, reclassification, and clinical utility, the findings address the practical consequences of decision-making in indeterminate thyroid disease. With further validation and refinement, this approach has the potential to support more individualized, proportionate, and patient-centered care in a clinical context long characterized by diagnostic uncertainty.

## Data Availability

The original contributions presented in the study are included in the article/**Supplementary Material**. Further inquiries can be directed to the corresponding author.
