# Supplementary material for: Clinical and ultrasound-based optimization of post-FNA management decisions in Bethesda III/IV thyroid nodules: a retrospective study
Source: Front Endocrinol (Lausanne). 2026 May 21;17:1807083. doi: 10.3389/fendo.2026.1807083 (PMC13233352; doi:10.3389/fendo.2026.1807083)
Supplement: Supplementary file 1 [file Table1.docx]

**Supplementary Table S1.** Missing data patterns across candidate variables

| Variable | Missing n (%) |
| --- | --- |
| FT4 | 38 (18.4%) |
| TSH | 34 (16.5%) |
| TPOAb | 52 (25.2%) |
| TgAb | 49 (23.8%) |
| HbA1c | 41 (19.9%) |
| LDL-C | 37 (18.0%) |
| Elastography category | 46 (22.3%) |
| Suspicious cervical lymph nodes | 21 (10.2%) |
| Echogenic foci classification | 18 (8.7%) |

Note: Missing data were primarily observed in laboratory variables and selected ultrasound descriptors that were not consistently recorded in routine clinical practice.

**Supplementary Table S2.** Comparison of selected baseline characteristics between complete-case and incomplete-case subsets

| Characteristic | Complete-case (n=121) | Incomplete-case (n=85) | *P* value |
| --- | --- | --- | --- |
| Female sex | 92 (76.0%) | 67 (78.8%) | 0.642 |
| Current smoking | 13 (10.7%) | 10 (11.8%) | 0.803 |
| Hypertension | 31 (25.6%) | 22 (25.9%) | 0.965 |
| Diabetes | 10 (8.3%) | 12 (14.1%) | 0.189 |
| Dyslipidemia | 26 (21.5%) | 21 (24.7%) | 0.594 |
| Multinodular goiter | 37 (30.6%) | 29 (34.1%) | 0.595 |
| Bethesda IV (vs III) | 51 (42.1%) | 32 (37.6%) | 0.517 |
| Taller-than-wide shape | 18 (14.9%) | 10 (11.8%) | 0.525 |
| Marked hypoechogenicity | 12 (9.9%) | 9 (10.6%) | 0.872 |
| Punctate echogenic foci | 28 (23.1%) | 22 (25.9%) | 0.652 |
| Suspicious cervical lymph nodes | 11 (9.1%) | 7 (8.2%) | 0.824 |
| Malignancy outcome | 52 (43.0%) | 35 (41.2%) | 0.803 |

Note: Complete-case analysis included patients with non-missing values for all predictors used in the multivariable model. Comparisons were performed using the chi-square test or Fisher’s exact test as appropriate. No statistically significant differences were observed between complete-case and incomplete-case subsets for the selected baseline characteristics or outcome distribution.
